# Bleeding in patients who underwent scheduled second-look endoscopy 5 days after endoscopic submucosal dissection for gastric lesions

**DOI:** 10.1186/s12876-018-0774-2

**Published:** 2018-04-10

**Authors:** Koichi Izumikawa, Masaya Iwamuro, Tomoki Inaba, Shigenao Ishikawa, Kenji Kuwaki, Ichiro Sakakihara, Kumiko Yamamoto, Sakuma Takahashi, Shigetomi Tanaka, Masaki Wato, Hiroyuki Okada

**Affiliations:** 1 Department of Gastroenterology, Kagawa Prefectural Central Hospital, 1-2-2 Asahi-machi, Takamatsu, Kagawa 760-8557 Japan; 2 Department of Gastroenterology and Hepatology, Okayama University Graduate School of Medicine, Dentistry and Pharmaceutical Sciences, 2-5-1 Shikata-cho, Kita-ku, Okayama, Okayama 700-8558 Japan; 3 Department of Internal Medicine, Fukuyama City Hospital, 5-23-1 Zao-cho, Fukuyama, Hiroshima 721-8511 Japan

**Keywords:** Antithrombotic treatment, Gastric neoplasms, Gastric ulcer, Second-look endoscopy, Prophylactic hemostasis

## Abstract

**Background:**

Bleeding after endoscopic submucosal dissection (ESD) in antithrombotic drug users is still one of the important issues to be solved. We performed scheduled second-look endoscopy (SLE) 5 days after ESD, when the resumption of antithrombotic agents is assumed to have achieved a steady state, rather than on the day after ESD. We investigated bleeding incidence and the status of ulcers.

**Methods:**

A total of 299 lesions in 299 patients subjected to ESD for gastric neoplasms were enrolled. A double dose of proton pump inhibitors was administered after ESD. SLE was planned 5 days after ESD. Post-ESD bleeding occurring before SLE was defined as early phase post-ESD bleeding, whereas bleeding after SLE was defined as later phase post-ESD bleeding. Forrest IIa and IIb ulcers are defined as high-risk ulcers requiring prophylactic hemostasis. We investigated risk factors for post-ESD bleeding, particularly focusing on the use of antithrombotic agents and the presence of high-risk ulcers requiring prophylactic hemostasis during SLE.

**Results:**

Under a double dose of proton pump inhibitors, early phase post-ESD bleeding occurred in 2.3% of non-users (5/218) and 6.2% of users of antithrombotic agents (5/81). High-risk ulcers were found in 19.0% of the cases during scheduled SLE (55/289). Later phase bleeding occurred in 5.5% of cases [2.8% of non-users (6/213) and 13.2% of users of antithrombotic agents (10/76)]. Cox regression analysis revealed that the risk factor for post-ESD bleeding was antithrombotic treatment (HR: 3.56; 95% CI: 1.63–8.02, *p* = 0.002) alone. Among patients with high-risk ulcers, a statistically significant increase in bleeding was observed in the later phase in patients under antithrombotic therapy, compared to those not receiving any antithrombotic agents (*p* = 0.001).

**Conclusions:**

Antithrombotic treatment is a risk factor for post-ESD bleeding despite SLE being scheduled 5 days after ESD. Later phase post-ESD bleeding was observed in 13.2% of the patients under antithrombotic treatment even after prophylactic hemostasis for high-risk ulcers.

**Trial registration:**

This study was registered in the UMIN Clinical Trials Registry System (000023306). Retrospectively registered on 23rd July 2016.

## Background

Although endoscopic submucosal dissection (ESD) has been established as a standard treatment for gastric neoplasms [1–3], post-ESD bleeding remains one of the various procedure-related adverse events that should not be overlooked [4, 5]. Known factors affecting the incidence of post-ESD bleeding include ESD technique, drugs administered prior to ESD (such as antiplatelet and anticoagulant drugs), gastric acid suppressing agents, and second-look endoscopy (SLE) [6–9]. In a recent study, we reported that vonoprazan, a potassium-competitive acid blocker, is superior to proton pump inhibitors (PPIs) in preventing post-ESD bleeding, based on a faster, stronger, and longer inhibition of gastric acid secretion than PPIs [10]. There is an increasing number of patients who are on antithrombotic agents and require ESD, since antithrombotic drugs are widely used for the treatment and prevention of thromboembolism. Thus, post-ESD bleeding in antithrombotic drug users is still one of the important issues to be solved.

For peptic ulcers with active bleeding, previous studies have revealed that SLE after initial endoscopic hemostasis improves mortality [11, 12]. SLE has been empirically performed after ESD with the expectation that it would be effective in reducing frequency of bleeding from ESD-induced ulcers as well as from peptic ulcers. SLE is typically performed on the day after ESD as post-procedural bleeding most frequently occurs within 24 h of ESD [5]. However, a consensus on the proper time of performing SLE has yet to be reached.

Currently, no evidence is available to support the usefulness of routine SLE in patients with hemorrhagic peptic ulcers, owing to recent improvements in endoscopic hemostatic devices and the introduction of PPIs [13]. Moreover there is accumulating evidence that SLE performed on the day after ESD does not help to decrease delayed bleeding from ESD-induced ulcers [14, 15]. However, the relationship between post-ESD bleeding incidence and high-risk ulcers that require prophylactic hemostasis, in antithrombotic drug users has not yet been fully investigated. In order to establish an appropriate strategy for management during the post-ESD period, further studies are required to address the role of specific interventions, such as the time of performing SLE, use of prophylactic hemostasis, and administration of acid-suppressing agents. It is important to accumulate data regarding post-ESD bleeding from manifold aspects.

It has been reported that suppression of platelet aggregation is stabilized 5 days after the administration of antiplatelet therapy such as low dose aspirin or clopidogrel, and anticoagulant therapy such as warfarin [16–18]. In our institution, we do not perform SLE on the day after ESD. Instead, we schedule SLE 5 days after ESD, when the antithrombotic effect is expected to be exhibited after resumption of antithrombotic drugs. The purposes of this study were i) to investigate the bleeding incidence and status of post-ESD ulcers and ii) to investigate risk factors for post-ESD bleeding.

## Methods

### Participants

Patients who underwent ESD for gastric neoplasm between January 2011 and March 2014 at Kagawa Prefectural Central Hospital, were retrospectively reviewed. In patients who underwent ESD more than twice, we analyzed only the initial lesions. Patient exclusion criteria for this study included the following: (i) patients in whom en bloc resection was not performed endoscopically; (ii) patients in whom endoscopic snare resection was performed; (iii) patients with a remnant stomach; (iv) patients who underwent ESD for lesions located in more than two regions of the stomach [upper (U), middle (M) or lower (L)] on the same day; and (v) patients in whom SLE was not performed 5 days after ESD. All patients enrolled in this study provided written informed consent before undergoing ESD. The association between post-ESD bleeding, state of ulcers during SLE according to Forrest classification, use of antithrombotic agents, and other clinical backgrounds was examined.

### Management of patients taking anticoagulants

Antithrombotic agents used in the study subjects were classified into antiplatelet agents (low-dose aspirin, cilostazol, ticlopidine, clopidogrel, icosapentate, sarpogrelate hydrochloride, beraprost sodium, limaprost alfadex, and dipyridamole) and anticoagulants (warfarin). Drug cessation periods before the ESD procedure were 5 days for low-dose aspirin, 7 days for thienopyridine derivatives (ticlopidine and clopidogrel), and 1 day for other antiplatelet agents. Warfarin was discontinued 3 days before ESD and heparin replacement was introduced. Unfractionated heparin (10,000 units/day) was administered and then discontinued 2 h before ESD. All antithrombotic agents were restarted 2 days after ESD, except in cases with post-ESD bleeding. Heparin replacement was discontinued when the prothrombin time-international normalized ratio reached to 1.50. SLE and prophylactic hemostasis were performed while on complete anticoagulant therapy.

Patients were divided into two groups according to their antithrombotic therapy at baseline: (1) the antithrombotic group, which included 81 lesions in patients who had been using antithrombotic agents before undergoing ESD; and (2) the non-antithrombotic group, comprising 218 lesions in patients who had not used antithrombotic agents.

### ESD procedures

ESD was conducted as one of the treatment options for lesions with a preoperative diagnosis of either a gastric adenoma or a possible node-negative early gastric cancer, based on the criteria proposed by Gotoda et al. [1, 19]. ESD was performed by one of the two board-certified endoscopists who had previously performed ESDs in > 200 gastric neoplasm cases. ESD was performed according to the standard ESD procedure, which consisted of the following: (i) marking a circumferential region around the lesion; (ii) submucosal injection of solution outside the marked region; (iii) mucosal incision outside the marked region; (iv) additional injection of 0.4% sodium hyaluronate with 0.1% epinephrine and 1% indigo carmine dye into the submucosa underneath the lesion; (v) submucosal dissection with insulated-tip knife-2 (KD-611 L, Olympus Medical Systems, Co., Tokyo. Japan); (vi) hemostasis of active bleeding and prophylactic coagulation of visible vessels on the mucosal defect performed with hemostatic forceps (Coagrasper®; Olympus Optical Co., Tokyo, Japan) in soft coagulation mode, both during submucosal dissection and at the final step of ESD; and finally (vii) retrieval of the specimen. An electrosurgical current was applied using an electrosurgical generator (VIO 300D; ERBE, Tübingen, Germany). The resected specimens were stretched, pinned flat on a corkboard, and measured.

### Management after ESD

A total of 40 mg/day omeprazole was intravenously infused into the patients on the day of ESD (day 0) and on the following day (day 1). Blood tests were performed 2 h after ESD and on day 0. Dietary intake was initiated and 20 mg/day of rabeprazole was administered daily, from day 2 in cases with no evidence of bleeding. Polaprezinc, which is commonly used in Japan for treating peptic ulcers [20, 21], was also administered from day 2. Blood tests and SLE were planned for day 5. Patients without post-ESD bleeding were discharged on that day. Rabeprazole and polaprezinc were administered to all patients until day 51 or day 52.

Post-ESD bleeding was defined as an episode of hematemesis and/or melena, or a decline in hemoglobin levels of ≥2 g/dL. Emergency endoscopy and endoscopic hemostasis were performed in patients with post-ESD bleeding. Post-ESD bleeding that occurred before SLE was defined as early phase post-ESD bleeding, whereas bleeding that occurred after SLE was defined as later phase post-ESD bleeding.

Emergency endoscopy was performed by board-certified endoscopists. Ulcers were classified according to the Forrest classification [22]. When adherent clots (IIb) were observed on the post-ESD ulcer during SLE, the endoscopist carefully checked whether non-bleeding visible vessels (IIa) existed after removing the clots [23]. When active bleeding (Ia and Ib) was observed on the post-ESD ulcer, endoscopic hemostasis with hemostatic forceps (Coagrasper®) in soft coagulation mode or argon plasma coagulation was performed.

### SLE

Patients were generally under strict supervision during the hospital stay after ESD and could be immediately treated for overt post-ESD bleeding. In addition, there was no available evidence regarding actual time of performing SLE and usefulness of prophylactic hemostasis for post-ESD ulcers for the prevention of post-ESD bleeding. Therefore, in accordance with the management strategy after endoscopic hemostasis of hemorrhagic peptic ulcers, we did not perform SLE on the day after ESD. Alternatively, we performed SLE on day 5 when the resumption of antithrombotic agents is assumed to achieve a steady state [16–18]. During SLE, post-ESD ulcers were classified according to the Forrest classification. Endoscopic treatment was performed with hemostatic forceps in soft coagulation mode or argon plasma coagulation for Forrest IIa and IIb ulcers, since these ulcers were considered to be at high risk of delayed bleeding. Therefore, Forrest IIa and IIb ulcers were defined as high-risk ulcers and Forrest IIc and III ulcers were classified as low-risk ulcers. The treatment for Forrest IIa and IIb ulcers during SLE, was prophylactic hemostasis. Forrest IIc and III ulcers were not treated during SLE.

### Analysis of gastric lesions

The morphology of the lesion was endoscopically classified as an elevated, flat, or depressed lesion. In cases wherein two resected lesions existed in the same region of the stomach, the lesion diameter was calculated as the sum of the diameters of the two lesions, and the morphology of the larger lesion alone was used for analysis. The depth of the lesion, and the presence or absence of ulcer scar within the lesion were pathologically diagnosed.

### Statistical analysis and ethical considerations

Continuous variables were expressed as the mean ± standard deviation. For statistical analysis, the chi square test was used for categorical data. Residual analysis was used to analyze the differences between the antithrombotic drugs. Univariate analysis was performed with the log-rank test. Cox regression analyses were performed for factors having statistical significance in univariate analysis. A Kaplan–Meier curve was generated, and a log-rank test was used to compare post-ESD bleeding incidences between groups. Differences with a *P*-value of < 0.05 were considered statistically significant. JMP Pro ver.10 (SAS Institute, Cary, NC, USA) software was used for statistical computations.

The present study was approved by the Clinical Ethics Committee of Kagawa Prefectural Central Hospital in accordance with the Helsinki Declaration, and was registered in the UMIN Clinical Trials Registry System (000023306).

## Results

Gastric ESD was performed for 403 lesions in 364 patients between January 2011 and March 2014. En bloc resection was achieved for 398 lesions (98.8%). Endoscopic snare resection was performed for 19 lesions, and 8 lesions were in patients with a remnant stomach. Twenty-four lesions were excluded from this study, since these lesions were resected during a second or third ESD. We further excluded 27 lesions, including two perforated lesions, which existed in more than two regions of the stomach. SLE was not performed 5 days after ESD for these lesions, because of ESD-related adverse events such as perforation (*N* = 2) and pneumonia (*N* = 2), and because day 5 was a non-working day of our hospital (*N* = 19). Among patients in whom SLE was not performed on day 5, 6 patients who were not on antithrombotic agents experienced post-ESD bleeding (two patients on day 6, three on day 7, one on day 8). Overall, we retrospectively analyzed 299 lesions in 299 patients in the present study.

Patients’ backgrounds are summarized in Table 1. Patients included 225 males and 74 females, with an average age of 71.6 ± 9.1 years. The 299 lesions consisted of 51 adenomas and 248 adenocarcinomas. The antithrombotic group included 81 lesions (27.1%), whereas the non-antithrombotic group was comprised of 218 lesions (72.9%). In the antithrombotic group, patients received a single antiplatelet agent (52 lesions), two or more antiplatelet agents (11 lesions), a single anticoagulant agent (12 lesions), or antiplatelet plus anticoagulant agents (6 lesions). One patient was on hemodialysis for chronic renal failure.

**Table 1:** Clinical characteristics of the gastric lesions

	Number	Percent
Total no. of gastric lesions	299	
Age (years, mean ± SD)	71.6 ± 9.1	
Sex		
Male	225	(75.3)
Female	74	(24.7)
Under antithrombotic therapy	81	(27.1)
Single antiplatelet agent	52	
≥ 2 antiplatelet agents	11	
Single anticoagulant agent	12	
Antiplatelet(s) plus anticoagulant	6	
On hemodialysis	1	(0.3)
Tumor location		
Upper third	47	(15.7)
Middle third	142	(47.5)
Lower third	110	(36.8)
Length of the resected specimen (mm, mean ± SD)	36.4 ± 15.3	
Morphology		
Elevated	174	(58.2)
Flat or depressed	125	(41.8)
Pathology		
Adenoma	51	(17.1)
Adenocarcinoma, differentiated type	231	(77.3)
Adenocarcinoma, undifferentiated type	17	(5.7)
Depth of invasion		
M	263	(88.0)
SM, ≤ 500 mm (micrometer)	16	(5.4)
SM, > 500 mm (micrometer)	20	(6.7)
Ulcer scar within the lesion	16	(5.4)
Curative resection by ESD	266	(89.0)

*SD* standard deviation, *M* mucosal layer, *SM* submucosal layer, *ESD* endoscopic submucosal dissection

**Fig. 1:**
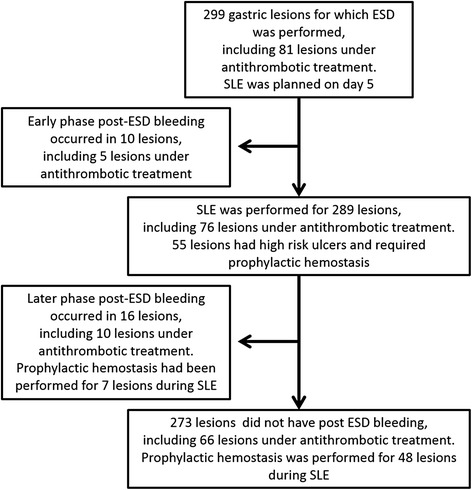
Flow chart of patients through the study. ESD, endoscopic submucosal dissection; SLE, second-look endoscopy

**Fig. 2:**
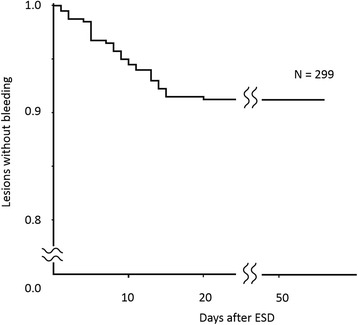
Time points and cumulative incidence of post-ESD bleeding. ESD, endoscopic submucosal dissection

Early phase post-ESD bleeding occurred in 10 lesions, 5 lesions were under antithrombotic treatment and the remaining 5 lesions were not under antithrombotic treatment. Later phase post-ESD bleeding occurred in 16 lesions, including 10 lesions under antithrombotic treatment. Consequently, post-ESD bleeding was detected in 26 lesions (8.7%; Fig. 1). Figure 2 shows the time points and cumulative incidence of post-operative bleeding. There were no instances of bleeding on day 21 or later.

Scheduled SLE was performed in 213 lesions in patients who were not receiving antithrombotic treatment and in 76 lesions in patients under antithrombotic treatment. Antithrombotic treatment included single antiplatelet treatment (*N* = 50, 65.8%), dual or more antiplatelet treatment (*N* = 11, 14.5%), single anticoagulant treatment (*N* = 12, 15.8%), and a combination of antiplatelet and anticoagulant treatment (*N* = 3, 3.9%). During SLE, the majority of the ESD-induced ulcers were Forrest IIc or III (*N* = 234, 81.0%). A total of 27 lesions (9.3%) were graded as Forrest IIa, while 28 lesions were classified as Forrest IIb (9.7%). Consequently, prophylactic hemostasis was performed for 55 lesions with high-risk ulcers (Forrest IIa and IIb ulcers, 19.0%). Procedures used for prophylactic hemostasis was argon plasma coagulation (*N* = 41) and coagulation by using hemostatic forceps (*N* = 14).

In high-risk ulcers, the ulcer diameter was ≥40 mm in 24/55 lesions (43.6%), compared with ≥40 mm diameter in 65/234 lesions (27.8%) with low-risk ulcers. The difference was statistically significant between the two groups with regard to the prevalence of ulcers measuring ≥40 mm (*p* < 0.034). Among the 55 patients with high-risk ulcers, 18 (32.7%) were under antithrombotic treatment, while among the 234 patients with low-risk ulcers, 58 (24.8%) were receiving treatment. The difference in the prevalence of antithrombotic treatment between the two groups was not statistically significant (*p* = 0.237) (Table 2).

**Table 2:** Ulcer status during second-look endoscopy according to the Forrest classification

	IIa + IIb (*N* = 55)	IIc + III (*N* = 234)	P* values
Diameter of the resected specimen			
< 40 mm	31 (56.4%)	169 (72.2%)	0.034
≥ 40 mm	24 (43.6%)	65 (27.8%)	
Antithrombotic agents			
Non-user	37 (67.3%)	176 (75.2%)	0.237
User	18 (32.7%)	58 (24.8%)	

Later phase post-ESD bleeding was observed in 16 lesions; 6/213 lesions without antithrombotic treatment (2.8%); 5/50 lesions under single antiplatelet treatment (10.0%); 2/11 lesions under dual or more antiplatelet treatment (18.2%); 1/12 lesion under single anticoagulant treatment (8.3%); and 2/3 lesions under a combination of antiplatelet and anticoagulant treatment (66.7%). Despite prophylactic hemostasis, overall 7/55 high-risk ulcers bled in the later phase (12.7%), compared to 9/234 low-risk ulcers that bled in the later phase (3.8%). The incidence of later phase bleeding from high-risk ulcers was significantly higher than that from low risk ulcers (*P* = 0.018). There was no statistically significant difference between lesions with regard to the use of prophylactic hemostasis, which included argon plasma coagulation (6/41, 14.6%) and hemostatic forceps (1/14, 7.1%) (*p* = 0.664). Among the 76 lesions in the antithrombotic group, later phase post-ESD bleeding occurred in 6/18 high-risk ulcers (33.3%) and in 4/58 low-risk ulcers (6.9%). Among the 213 lesions in the non-antithrombotic group, later phase post-ESD bleeding occurred in 1/37 high-risk ulcers (2.7%) and in 5/176 low-risk ulcers (2.8%).

**Fig. 3:**
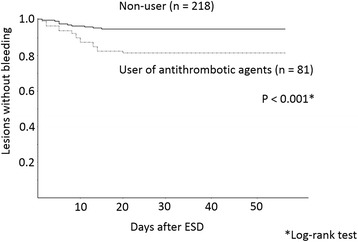
Time points and cumulative incidence of post-ESD bleeding in users and non-users of antithrombotic agents. ESD, endoscopic submucosal dissection

Univariate analysis of all 26 lesions with post-ESD bleeding (early phase plus later phase post-ESD bleeding) revealed that a diameter of the resected specimen ≥40 mm (*p* = 0.036) and use of antithrombotic agents (*p* < 0.001) were statistically significant factors for bleeding (Table 3). We used the diameter of the resected specimen ≥40 mm and antithrombotic treatment before undergoing ESD in Cox regression analysis, which revealed that the risk factor for post-ESD bleeding was an antithrombotic treatment (HR: 3.56; 95% CI: 1.6–8.02; *p* = 0.002) alone. Figure 3 shows the Kaplan-Meier curve of post-ESD bleeding in patients under antithrombotic treatment and patients without antithrombotic treatment.

**Table 3:** Univariate analyses of post-ESD bleeding risk

	Early phase post-ESD bleeding				Later phase post-ESD bleeding				Total instances of post-ESD bleeding				
	Bleeding		Non-bleeding		Bleeding		Non-bleeding		Bleeding		Non-bleeding		*P*-value
No. of lesions	10		289		16		273		26		273		
Aged ≥65 years	9	90.0%	231	79.9%	13	81.3%	218	79.9%	22	84.6%	218	79.9%	0.539
Male sex	9	90.0%	216	74.7%	14	87.5%	202	74.0%	23	88.5%	202	74.0%	0.103
Tumor location													
Upper third	4	40.0%	43	14.9%	2	12.5%	41	15.0%	6	23.1%	41	15.0%	0.259
Middle third	3	30.0%	139	48.1%	10	62.5%	129	47.3%	13	50.0%	129	47.3%	0.809
Lower third	3	30.0%	107	37.0%	4	25.0%	103	37.7%	7	26.9%	103	37.7%	0.277
Length of the resected specimen ≥40 mm	6	60.0%	89	30.8%	7	43.8%	82	30.0%	13	50.0%	82	30.0%	0.036
Elevated type morphology	6	60.0%	168	58.1%	9	56.3%	159	58.2%	15	57.7%	159	58.2%	0.949
Depth of invasion													
M	8	80.0%	255	88.2%	13	81.3%	242	88.6%	21	80.8%	242	88.6%	0.230
SM, ≤500 uL	1	10.0%	15	5.2%	1	6.3%	14	5.1%	2	7.7%	14	5.1%	0.562
SM, > 500 uL	1	10.0%	19	6.6%	2	12.5%	17	6.2%	3	11.5%	17	6.2%	0.297
Ulcer scar within the lesion	0	0.0%	16	5.5%	0	0.0%	16	5.9%	0	0.0%	16	5.9%	0.214
Use of antithrombotic agents	5	50.0%	76	26.3%	10	62.5%	66	24.2%	15	57.7%	66	24.2%	0.0002
Forrest IIa + IIb ulcers requiring prophylactic hemostasis	–		–		7	43.8%	48	17.6%	7	26.9%	48	17.6%	0.275

*ESD* endoscopic submucosal dissection, *CI* confidence interval, *M* mucosal layer, *SM* submucosal layer

Post-ESD bleeding was observed in 11/218 lesions without antithrombotic treatment (5.0%). Among them, early phase post-ESD bleeding occurred in 5/218 lesions (2.3%) and later phase post-ESD bleeding occurred in 6/213 lesions (2.8%). Conversely, in lesions under antithrombotic treatment, post-ESD bleeding arose in 15/81 lesions (18.5%); where bleeding occurred in the early phase in 5/81 lesions (6.2%) and in the later phase in 10/76 lesions (13.2%).

**Fig. 4:**
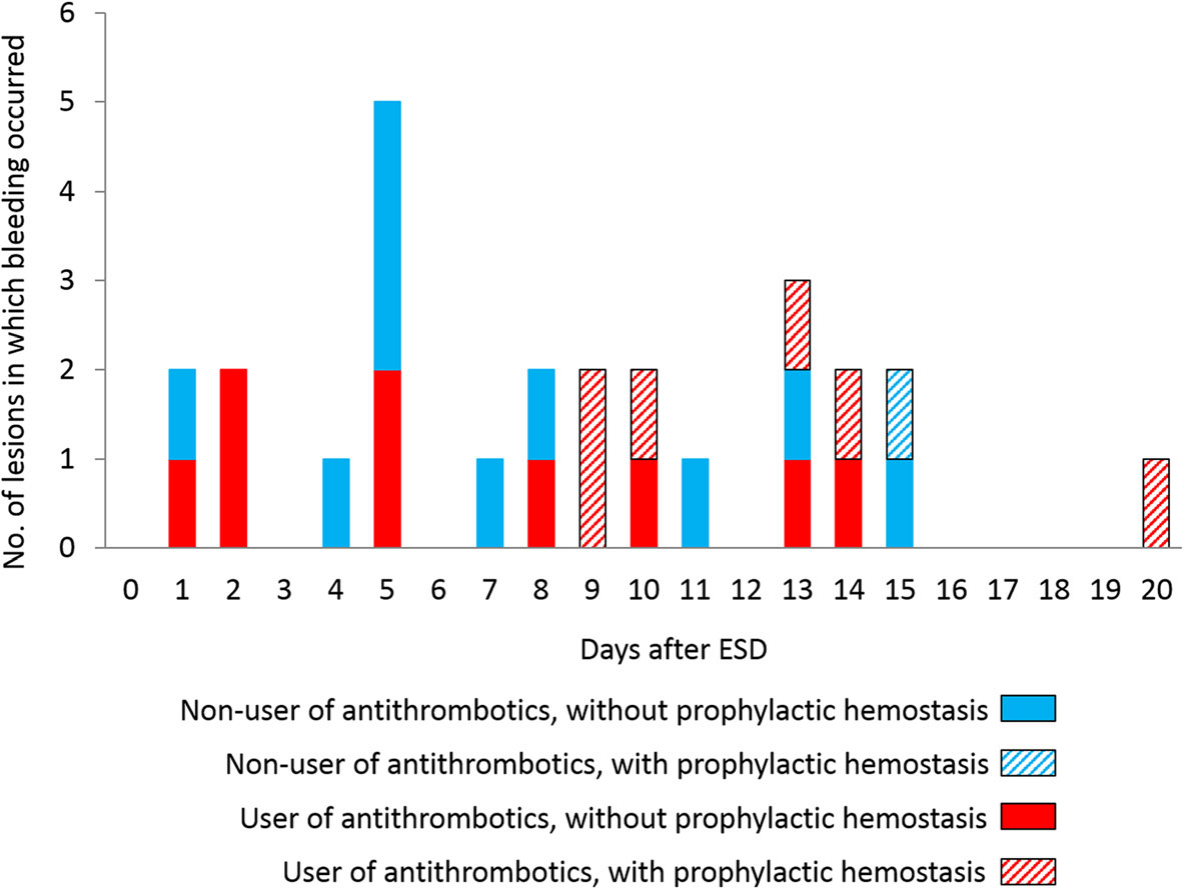
Schematic diagram showing the number of patients with post-ESD bleeding and the time elapsed since their ESD procedure

Figure 4 shows the time points when post-ESD bleeding was detected, according to user or non-user of antithrombotic agents and presence or absence of prophylactic hemostasis. Non-scheduled endoscopic examinations were performed in 10 patients with early phase post-ESD bleeding, in whom Forrest Ia (*N* = 2), Ib (*N* = 5), IIa (*N* = 1), and III (*N* = 2) ulcers were identified. Hemostasis was performed for Forrest Ia and Ib ulcers with hemostatic forceps. Argon plasma coagulation was used for Forrest IIa ulcers. No endoscopic procedure was done for Forrest III ulcers. These patients were excluded from the analysis for scheduled SLE. Two patients exhibited melena and three patients showed a decline in hemoglobin levels by ≥2 g/dL, 5 days after ESD. These patients were classified as early phase post-ESD bleeding. There were approximately equal proportions of early phase post-ESD bleeding (10/299, 3.3%) and later phase post-ESD bleeding complications (16/289, 5.5%).

**Fig. 5:**
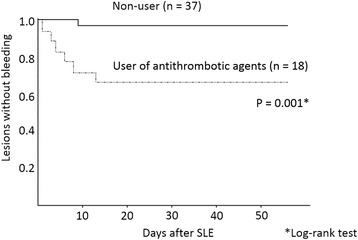
Time points and cumulative incidence of later phase post-ESD bleeding that occurred after prophylactic hemostasis in patients with high-risk ulcers (Forrest IIa and IIb ulcers). ESD, endoscopic submucosal dissection; SLE, second-look endoscopy

Log-rank tests revealed that bleeding incidence after SLE was higher in high-risk ulcers in the antithrombotic group, compared with those in the non-antithrombotic group. The result was statistically significant (*p* = 0.001) (Fig. 5).

Later phase post-ESD bleeding occurred in seven high-risk ulcers. Emergency endoscopy and endoscopic hemostasis were performed in 5/7 high-risk ulcers with later phase post-ESD bleeding. The remaining two patients were treated with medication only, as they rejected emergency endoscopic examination. In four of five bleeding high-risk ulcers, bleeding was observed from the sites where prophylactic hemostasis was performed during SLE. In this study, all patients with post-ESD bleeding recovered and were discharged from our hospital.

## Discussion

Although the reported frequency of post-ESD bleeding varies depending on the definition of “bleeding”, it has been reported as ~ 5%, provided that post-ESD bleeding is defined as an episode of hematemesis/melena, or a decline in hemoglobin levels by ≥2 mg/dL, as seen in the present study [4, 8]. In this study, post-ESD bleeding was observed in 8.7% of lesions, which was relatively higher than the reported incidence. In this study, 27.1% of the lesions (81/299 lesions) had been treated with antithrombotic agents before undergoing ESD. Therefore as compared to other studies (8.0-16.3%), the relatively higher number of patients under antithrombotic therapy enrolled for this study may have resulted in a higher incidence of post-procedural bleeding.

In this study, the incidence of post-ESD bleeding in the early and later phase were 2.3 and 2.8%, respectively, in the non-antithrombotic group, and 6.2 and 13.2%, respectively, in the antithrombotic group. In a previous randomized controlled study, in which all participants were non-users of antithrombotic agents and did not undergo SLE on the day after ESD, post-ESD bleeding occurred in the early phase in 2.3% of cases [24], which is similar to the incidence rate in our study. The present study also revealed that in the antithrombotic group, post-ESD bleeding occurred more frequently in the later phase than early phase (13.2% vs 6.2%). We speculated that bleeding is less likely to occur in the early phase because several days are required after resumption of the antithrombotic agents for the drug efficacy to reach a steady state [16–18]. Another possible reason is damage to the post-ESD ulcer during SLE, owing to hemostatic procedures and/or endoscopic examination itself.

Koh et al. reported that antithrombotic agents increase post-ESD bleeding incidence ≥5 days after ESD, whereas these agents did not increase post-ESD bleeding during the 0–4 days after ESD [7]. In addition, it has been reported that heparin replacement after the discontinuation of antithrombotic agents is a risk factor for post-ESD bleeding ≥5 days after ESD [25].

Recent studies comparing between patients undergoing prophylactic hemostasis and patients without prophylactic hemostasis during SLE performed on the day after ESD reported that although there was no statistical difference, post-ESD bleeding incidence relatively increased in the former patient group [15, 24], suggesting the possibility that prophylactic hemostasis did not prevent post-ESD bleeding. Mochizuki et al. speculated that prophylactic hemostasis itself causes additional damage to the vessels within the ESD-induced ulcer and may induce hemorrhage [24]. We were unable to evaluate role of prophylactic hemostasis in this study, as it was applied for all high-risk ulcers (Forrest IIa and IIb ulcers).

Coagulation is used as a hemostatic procedure to prevent bleeding during both ESD and SLE. Argon plasma coagulation and hemostatic forceps were used in this study. The safety of these procedures during ESD has been previously reported [8, 26]. However, thermal injury from argon plasma coagulation increases depending on the energy output [27]. For example, application of argon plasma coagulation for intact colon mucosa can contribute to muscle layer damage [28]. During SLE, thermal damage by argon plasma coagulation and hemostatic forceps followed by exposure to gastric acid may injure the arteries. This hypothesis is supported by the present results, which indicated that later phase bleeding in high-risk ulcers occurred where prophylactic hemostasis was performed.

Typically, endoscopic hemostasis is performed for Forrest IIa and IIb ulcers. Therefore, the true incidence rate of hemorrhage from Forrest IIa and IIb ulcers has not been revealed till date. Kim et al. performed scheduled SLE on the day after ESD and investigated post-ESD bleeding incidence according to each Forrest classification in a patient population, of which 11.2% were under antithrombotic treatment [15]. In patients who did not undergo prophylactic hemostasis, bleeding occurred in 11.1% of Forrest IIa ulcers and 12.1% of Forrest IIb ulcers. In patients who underwent prophylactic hemostasis, bleeding occurred in 20.0% of Forrest IIa ulcers and 21.4% of Forrest IIb ulcers. Overall bleeding incidence in Forrest IIa and IIb ulcers without prophylactic hemostasis was 11.8%, and in Forrest IIa and IIb ulcers with prophylactic hemostasis was 20.5%. Kim et al. speculated that air insufflations and the hemostatic procedure during SLE may have caused tissue injury and exposed arteries, resulting in delayed bleeding. In this context, SLE and prophylactic hemostasis may not be necessary to prevent post-ESD bleeding.

Differences in bleeding incidence rates of Forrest IIa and IIb ulcers with prophylactic hemostasis between our study (12.7%) and the study by Kim et al. (20.5%) may be explained by the different times of performing SLE and prophylactic hemostasis. Kim et al. performed SLE on the day after of ESD, whereas SLE was performed on day 5 in the present study. ESD-induced gastric ulcers are reported easier and faster to heal, compared with peptic ulcers [29]. Therefore, since the healing process of ulcers is likely to progress by day 5, hemostatic procedure may be less harmful for post-ESD ulcers when SLE is performed on day 5.

A recent meta-analysis has reported that delayed post-ESD bleeding was more frequent in patients who underwent prophylactic hemostasis than in those who did not [30]. However, previous studies were single-arm studies, in which prophylactic hemostasis was intended for all high-risk ulcers, and the relationship between antithrombotic agents and prophylactic hemostatic procedure has never been taken into consideration.

The bleeding incidence and the status of post-ESD ulcers have never been investigated in users and non-users of antithrombotic agents, who do not undergo SLE on the day after ESD. The present study revealed that under a double dose of PPIs, early phase post-ESD bleeding occurs in 2.3% of non-users and in 6.2% of users of antithrombotic agents. We also demonstrated that high-risk ulcers were found in 19.0% of the cases during scheduled SLE, 5 days after ESD.

We consider that a possible alternative of SLE may be only to perform emergency endoscopic examination in patients exhibiting post-ESD bleeding. Administration of vonoprazan may be another alternative of SLE, which enables stronger and faster suppression of gastric acid secretion [10].

This study had several limitations. First, the sample size was relatively small. Second, this study was retrospectively conducted in a single institution. Third, in cases with two lesions resected by ESD, although the resected areas of the two lesions were close to each other, the actual diameter of the post-ESD ulcer created by ESD was not identical to the calculated value as the sum of the diameters of the two lesions. Fourth, there mights have been technical disparities in the endoscopic hemostatic procedures conducted by the endoscopists. Fifth, none of the included patients were on newer anticoagulant agents, that is, direct oral anticoagulant. Thus, further multi-centered studies with larger sample sizes are required.

## Conclusions

In conclusion, we performed SLE 5 days after ESD, when the resumption of antithrombotic agents is assumed to achieve a steady state, rather than on the day after ESD. We investigated the bleeding incidence and the status of ulcers, and the results revealed that the (i) use of antithrombotic agents before ESD was a risk factor for post-ESD bleeding, (ii) post-ESD bleeding occurred in 3.3% of cases during the early phase (2.3% of non-users and 6.2% of users of antithrombotic agents), (iii) high risk ulcers were found in 19% of the cases during scheduled SLE, and (iv) even after prophylactic hemostasis for a high-risk ulcer (Forrest IIa or IIb ulcer), bleeding occurred in 5.5% of cases during the later phase (2.8% of non-users and 13.2% of users of antithrombotic agents). Consequently, healthcare providers must be particularly aware of bleeding from high-risk ulcers in patients under antithrombotic treatment, irrespective of prophylactic hemostasis during ESD. The data obtained in this study will serve as useful reference for future research.

## Abbreviations

ESD: Endoscopic submucosal dissection
PPIs: Proton pump inhibitors
SLE: Second-look endoscopy
